# *Mycobacterium tuberculosis* Beijing Genotype Resistance to Transient Rifampin Exposure

**DOI:** 10.3201/eid2011.130560

**Published:** 2014-11

**Authors:** Alice L. den Hertog, Sandra Menting, Dick van Soolingen, Richard M. Anthony

**Affiliations:** KIT Biomedical Research, Royal Tropical Institute (KIT), Amsterdam, the Netherlands (A.L. den Hertog, S. Menting, R.M. Anthony);; National Institute for Public Health and the Environment (RIVM), Bilthoven, the Netherlands (D. van Soolingen);; Radboud University Medical Center, Nijmegen, the Netherlands (D. van Soolingen)

**Keywords:** tuberculosis, MDR TB, Beijing genotype strains, EAI genotype strains, H37Ra, antituberculosis drugs, rifampin, microcolony growth monitoring, Mycobacterium tuberculosis, tuberculosis and other mycobacteria, bacteria, antimicrobial resistance

**To the Editor:** We read with interest the discussion between de Steenwinkel et al. and Werngren (*1*) regarding the high mutation frequencies de Steenwinkel et al. reported for some *Mycobacterium tuberculosis* strains (*2*). We investigated the effect of rifampin exposure on the growth of these strains in detail.

We share Werngren’s surprise at the extraordinary mutation frequencies observed for the Beijing strains. We previously estimated the mutation rate of 1 of the strains tested at ≈1 × 10^–7^ (*3*), which is within the range typically reported for acquisition of rifampin resistance by strains from Beijing and other lineages of *M. tuberculosis* (*4*,*5*). Several explanations were proposed for the detection of a high frequency of mutants on rifampin exposure, mainly related to methods or experimental variation (*1*). We propose an alternative interpretation of these findings on the basis of new experimental data (Figure). 

**Figure:**
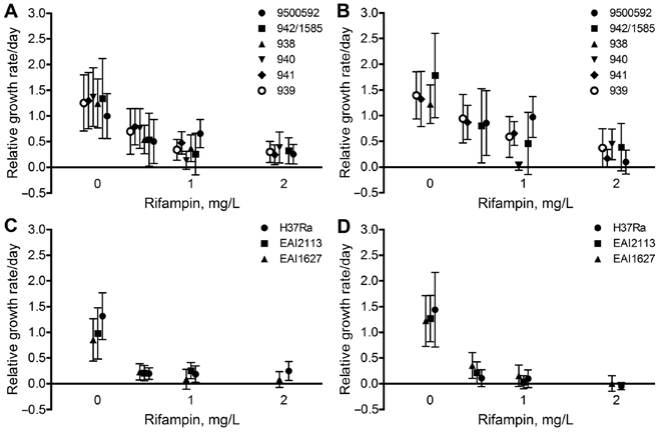
Eight-day-old microcolonies (≈10^2^ cells per colony) of a panel of *Mycobacterium tuberculosis* Beijing strains (A, B) and East African Indian strains and strain H37Ra (C, D). Growth of the strains was monitored after 4 hours of exposure to different concentrations of rifampin. For all colonies, the growth rate relative to preexposure growth rate was calculated at 0–1 days after exposure for a median of 889.5 (interquartile range 478.75–1611.25) colonies (left panels) and 1–5 days after exposure for a median of 363 (interquartile range 241.25–843.00) colonies (right panels) per strain and per condition. Plots are averages ±SD (indicated by error bars). The experimental conditions failed to totally inhibit growth of most Beijing colonies even at 5 days postexposure, whereas for the non-Beijing strains, virtually no growth was detectable at 5 days postexposure.

These data show that some *M. tuberculosis* strains persist longer than others after transient exposure to low concentrations of rifampin, which could create a wider window for the (possibly stimulated) generation and selection of resistant mutants. Thus, the frequency of 10^–3^ resistant CFUs selected on rifampin may represent not the frequency of preexisting mutants but rather frequency of mutants generated by a population of persisting (stressed) cells during exposure to low levels of antimicrobial drugs. We investigated the effect of transient exposure to rifampin on colony growth rate and post–antimicrobial drug outgrowth on a panel of Beijing and non-Beijing *M. tuberculosis* strains, including some studied in the work of Steenwinkel et al. (*2*). We used a culture method developed on the basis of den Hertog et al. (*6*) but with higher throughput and improved imaging and analysis, in which individual colonies are monitored over time and growing colonies can be moved from 1 solid medium to another. 

We injected ≈5 × 10^4^ CFUs of *M. tuberculosis* per cm^2^, consisting mostly of single cells (*6*), on porous aluminum oxide supports (PAOs) on MB7H11 agar + OADC (Becton Dickinson, Sparks, MD, USA). After 8 days’ incubation at 36°C, the PAOs containing microcolonies consisting of ≈200 cells were moved onto medium containing 0.5–2 μg/mL of rifampin for 4 h, after which the PAOs were returned to nonselective medium. Filters were monitored for colony growth by using a MuScan microscopic system (LumiByte BV, Nuenen, the Netherlands) at 5× magnification; a ≈0.44-cm^2^ area was imaged at specific intervals before and after exposure to rifampin. We used Fiji (http://fiji.sc/Fiji) and in-house software (*6*) to extract the surface areas of all identified colonies at all available time points; individual growth rates could thus be calculated for all colonies. In total, 36,408 colonies were defined as objects with a growth rate of >20% from days 6 and 8 after inoculation with >0.5 circularity.

As shown in the Figure, the growth of non-Beijing strains studied (East African/Indian strains and strain H37Ra) was almost completely inhibited during the first 24 hours after rifampin exposure. In contrast, all the Beijing genotype strains studied showed residual growth in most colonies in the first 24 hours after rifampin exposure. At 1–5 days postexposure, the pattern remained the same; the Beijing strain colonies were more capable of persisting and exhibited slow but sustained growth after exposure to low concentrations of rifampin. To confirm that this effect was a result of persistence rather than generation of resistant mutants, we transferred the colonies growing after transient rifampin exposure of Beijing strain 1585 to a medium containing 8 mg/L rifampin. Their growth was completely inhibited, and molecular analysis did not detect any of the most prevalent rifampin resistance–associated mutants (data not shown).

We believe that these results provide a possible explanation for the otherwise unrealistically high (apparent) mutation frequency reported by de Steenwinkel et al. (*2*). If these strains are capable of persisting at low concentrations of rifampin, this extended period would provide a window for the generation of mutants during or after exposure. Stress may also play a role; *rpoB* gene mutants have shown to exhibit a stringent-like response (*7*), and defective *rpoB* activity as a result of low-level rifampin exposure could induce a similar response. If rifampin induces a stress response, the situation may be analogous to the high mutation rates seen after quinolone exposure (*8*).

In summary, our data show that the high apparent *M. tuberculosis* strain mutation frequency reported by de Steenwinkel et al. (*2*) may be a result of the higher tolerance to rifampin of some Beijing strains. This tolerance likely results in a specific window of rifampin concentrations that, possibly combined with subsequent error-prone replication/outgrowth, enables the generation and selection of new mutants, rather than the selection of preexisting mutants. When interpreted in the light of our observations, the unexpected results of de Steenwinkel et al. could help explain the association of Beijing genotype strains with drug resistance and relapse (*9*,*10*). Drug levels achieved during treatment may be much more critical in preventing the accumulation of rifampin-resistant mutants for these strains than for other genotypes.
